# Vasohibin-1 is identified as a master-regulator of endothelial cell apoptosis using gene network analysis

**DOI:** 10.1186/1471-2164-14-23

**Published:** 2013-01-16

**Authors:** Muna Affara, Debbie Sanders, Hiromitsu Araki, Yoshinori Tamada, Benjamin J Dunmore, Sally Humphreys, Seiya Imoto, Christopher Savoie, Satoru Miyano, Satoru Kuhara, David Jeffries, Cristin Print, D Stephen Charnock-Jones

**Affiliations:** 1 Department of Obstetrics and Gynaecology, University of Cambridge, The Rosie Hospital, Robinson Way, Cambridge, CB2 0SW, U.K; 2 Systems Pharmacology Research Institute, GNI Ltd., Fukuoka, Japan; 3 Human Genome Centre, Institute of Medical Science, The University of Tokyo, Tokyo, Japan; 4 Graduate School of Genetic Resources Technology, Kyushu University, Fukuoka, Japan; 5 MRC, The Gambia Unit, Fajara, The Gambia; 6 Department of Molecular Medicine and Pathology, School of Medical Sciences, University of Auckland, Auckland, New Zealand; 7 Bioinformatics Institute, University of Auckland, Auckland, New Zealand; 8 National Institute for Health Research, Cambridge Comprehensive Biomedical Centre, Cambridge, UK

**Keywords:** Vasohibin, HUVEC, Bayesian, Gene regulatory network

## Abstract

**Background:**

Apoptosis is a critical process in endothelial cell (EC) biology and pathology, which has been extensively studied at protein level. Numerous gene expression studies of EC apoptosis have also been performed, however few attempts have been made to use gene expression data to identify the molecular relationships and master regulators that underlie EC apoptosis. Therefore, we sought to understand these relationships by generating a Bayesian gene regulatory network (GRN) model.

**Results:**

ECs were induced to undergo apoptosis using serum withdrawal and followed over a time course in triplicate, using microarrays. When generating the GRN, this EC time course data was supplemented by a library of microarray data from EC treated with siRNAs targeting over 350 signalling molecules.

The GRN model proposed *Vasohibin-1* (*VASH1*) as one of the candidate master-regulators of EC apoptosis with numerous downstream mRNAs. To evaluate the role played by *VASH1* in EC, we used siRNA to reduce the expression of *VASH1.* Of 10 mRNAs downstream of VASH1 in the GRN that were examined, 7 were significantly up- or down-regulated in the direction predicted by the GRN.Further supporting an important biological role of *VASH1* in EC, targeted reduction of *VASH1* mRNA abundance conferred resistance to serum withdrawal-induced EC death.

**Conclusion:**

We have utilised Bayesian GRN modelling to identify a novel candidate master regulator of EC apoptosis. This study demonstrates how GRN technology can complement traditional methods to hypothesise the regulatory relationships that underlie important biological processes.

## Background

The explosion of systems biology in recent years, facilitated by sequencing of the human genome [1,2] and the development of high throughput methods to rapidly characterise and quantify biological systems [3-6], has promoted understanding of complex biological and pathological processes. Gene regulatory networks (GRN) represent a systems biology approach, taking advantage of the growing number of RNA abundance data sets generated by modern high throughput methods such as microarrays or RNAseq, to holistically model interactions between molecules in cells and tissues. GRN are usually displayed as directed graphs - *nodes* represent mRNA abundance and *edges* represent some form of regulatory relationship between the nodes. The reverse engineering of GRN from gene expression data has been used to understand molecular interactions in both bacterial and lower eukaryotic organisms, as well as in more complex mammalian systems. GRN employ simple correlation [7] or Boolean [8] methods, algorithms based on mutual information [9,10] as well as Bayesian methods. Computational frameworks have been generated to simultaneously perform several types of GRN analysis [11]. Bayesian GRN are in theory especially powerful for inference of *causal* relationships between mRNAs in noisy microarray data [12,13]. In Bayesian GRN, the probability of the abundance of each mRNA node is modelled using a function that takes as its inputs the abundance of the node's parent mRNAs. The edges in a Bayesian GRN can represent hidden protein, non-coding RNA or metabolite-based regulatory relationships [14]. Therefore, Bayesian GRN can in theory capture information about a subset of the complex cellular regulatory circuitry [15]. Many GRN developed to date have had a ‘scale free’ structure [10,16], in which a small number of “hub” RNAs can be identified that are connected to large numbers of downstream RNAs in the networks. These hub RNAs are candidate master-regulators of transcription and other cellular processes. Their identification is based on relationships in the data between the hub RNA and their downstream RNAs in the GRN structure, which are usually referred to as "children". Therefore, the amount of data behind the identification of hub RNAs is much greater than the amount of data behind the identification of individual edges, and correct identification of hubs may be easier in theory than the correct identification of individual edges.

Apoptosis is pivotal for normal EC function [17], and the dysregulation of endothelial apoptosis is a key step in the development of numerous pathologies [18], including cardiovascular disease [19-21] and tumourogenesis [22-25]. Understanding the regulatory events occurring during this process in a holistic manner may provide insight into normal vascular development and maintenance, as well as vascular pathologies. Although there has been extensive characterisation of the EC proteins involved in apoptotic pathways [26-28], there have been fewer investigations into regulation of the transcriptome in ECs undergoing apoptosis [17,29].

To begin to address this issue, our group have previously used Bayesian GRN to identify molecular interactions involved in survival factor deprivation (SFD)-induced EC apoptosis and cell cycle arrest [18]. This previous study used micorarray data over a triplicated eight time point SFD time course. Previous studies have illustrated the value of supplementing time series data with gene disruption data (e.g. [30]). Since at the time we were especially interested in regulation of the cell cycle, in this previous work the time series data was supplemented by eight microarrays from EC cultures treated with siRNAs targeting molecules associated with the cell cycle. This analysis identified several GRN master regulator RNAs including the *γ-amino butyric acid (GABA)-Receptor Associated Protein* (*GABARAP*) [18].

In theory, the greater the volume of high-quality siRNA data used to supplement time course data, and the broader the range of RNAs targeted by the siRNAs, the more likely it is that accurate predictions can be made by GRN. Therefore, in this current study we have expanded our previous analysis by combining triplicated eight time point SFD time course data with a much larger library of EC siRNA disruptant microarray data, which was generated from the knockdown of 351 different mRNA transcripts that encode proteins with a broad range of functions in EC [11]. This expanded analysis identified numerous GRN master regulators, many of which were already known to play important roles in EC biology. However, we noted one major master regulator RNA named *Vasohibin-1* (*VASH1*) that had not at the time been extensively studied in EC apoptosis. Therefore, we investigated the function of *VASH1* in regulating mRNA abundance and in the process of EC apoptosis. We targeted *VASH1* using siRNA and then used quantitative polymerase chain reaction (PCR) to examine the abundance of 10 of the 31 mRNAs directly downstream of *VASH1* in the GRN. 7 of these 10 mRNAs were significantly up- or down-regulated in the direction predicted by the GRN when *VASH1* expression was reduced. We also show that *VASH1* is required for the apoptotic response in EC treated with SFD.

## Methods

### Cell culture and siRNA transfection

Umbilical cords were collected with written informed maternal consent and the approval of the Cambridge (UK) Research Ethics Committee. Human Umbilical Vein ECs (HUVECs) were isolated by collagenase digestion, as previously described [31]. Cells were cultured in fully supplemented media without antibiotics (basal EBM-2 with a propriety mix of heparin, hydrocortisone, epidermal growth factor, fibroblast growth factor, vascular endothelial growth factor, 2% foetal calf serum (FCS) Lonza, Cambridge, UK), at 37°C/5% CO_2_. To carry out siRNA transfection, HUVEC pools consisting of 10 biological isolates (of equal contribution) were prepared using passage 3 cultured cells. The HUVEC pools were plated in 6-well plates at 2.5 × 10^5^ per well and left for 24hrs until approximately 70% confluent. siRNA transfection was carried out using pools of four siRNA duplexes from Dharmacon Inc (Lafyette, Colorado, USA) and the SiFectamine transfection reagent (ICVEC, London, UK) according to the manufacturer’s instructions.

### RNA processing and microarray preparation

RNA was extracted using TRIzol® reagent (Invitrogen, London, UK). RNA quality was assessed using the Agilent 2100 bioanalyser. Biotin labelled cRNA was generated and hybridised on the CodeLink Human Uniset 20K microarrays following the manufacturer’s instructions (Applied Microarrays, Tempe, Arizona, USA, formally supplied by GE Healthcare).

### Quantitative PCR

cDNA was synthesised from 1μg of total RNA using the QuantiTect reverse transcription kit (Qiagen, Crawley, UK), following the manufacturers protocol. Quantitative PCR was carried out using an ABI 7700 sequence analyser (Applied Biosystems, Warrington, UK). Reactions were performed using the Applied Biosystems universal master mix according to the manufacturers instructions. The Taqman probe / primers used were: *VASH1* (Hs00208609_m1), *SOX18* (Hs00746079_s1), *PTX3* (Hs00173615_m1), *FAM78A* (Hs00604618_m1), *PPARA* (Hs00231882_m1), *SLC7A2* (Hs00952727_m1), *BDNF* (Hs00542425_s1), *MTSS1* (Hs00207341_m1), *BTG2* (Hs00198887_m1), *TNFSF-12* (Hs00356411_m1), *FLT4* (Hs01047677_m1) and *NTRK2* (Hs00178811_m1), all from Applied Biosystems.

### Previosuly generated microarray datasets used in this study

The siRNA targeting of 351 different mRNA transcripts, chosen for their importance in EC biology, including transcription factors, signalling molecules, receptors and ligands is described by Hurley et. al. [11]. The microarray data from these 351 siRNA experiments is available from Gene Expression Omnibus, reference GSE27869.

The generation of triplicated microarray data from an eight time point HUVEC SFD time course has been described previously [18]. Briefly, HUVEC RNA was extracted at time points 0, 0.5, 1.5, 3, 6, 9, 12 and 24 hours after survival factor withdrawal (i.e. transfer from complete media to basal EBM-2 media with no supplements apart from 2% charcoal stripped serum), and hybridised onto CodeLink Human Uniset 20K microarrays. The raw and normalised triplicate time course microarray data has been deposited in Gene Expression Omnibus, accession number GSE23067.

### Data processing

CodeLink microarray quality was assessed using the CodeLink Expression analysis software v4.0. The array data were filtered to remove probes that did not contain “Good” flags in 90% of the arrays, as measured by the CodeLink Expression analysis software. The log base2 (log2)–transformed apoptosis time course data and 351 siRNA disruptant data were then both normalised using the Loess method [32,33]. For the disruptant dataset log2 ratios against a virtual median array were calculated and these ratios were then z-transformed within each microarray prior to network inference.

For the SFD time course data, we selected transcripts concordantly regulated in abundance across the timecourse to used for GRN generation as previously described [18]. Briefly, log ratios between each time point and the first time point were calculated for all transcripts. For each transcript at each time point z-scores were then calculated by subtracting the log2 ratios from the mean of log2 ratios for that time point, and dividing by the standard deviation of log2 ratios for that time point. Transcripts were then selected that had −2 ≤ z ≤+2 at ≥ *two* adjacent time points in the triplicate data set. This analysis was repeated using the last time point instead of the first time point, and the union of the RNA lists prodced by the analyses that used the first and last timepoints was taken as the final list of concordantly expresed RNAs. For comparison to this z-score method, ANOVA was used to identify RNAs significantly differntially expressed at *two* adjacent time points relative to either the first or last time point, and the empirical Bayes method of Tai and Speed [34] was also applied. In addition, a statistically more complex method was used to identify RNAs significantly differntially expressed across the timecourse; generalised estimating equations with a Markov correlation model were fitted to the timecourse data. Contrasts were used to identify linear relationships and quadratic trends within the data using Matlab's GEEQBOX toolbox (http://www.mathworks.com/products/matlab/). Thresholds for concordant regulation were set using an absolute linear coefficient of >21 (and linear q value <0.01) OR an absolute quadratic coefficient of > 7 (and q value <0.01).

All other bioinformatic manipulations used the R software package, (http://www.R-project.org), and unless otherwise stated, multiple testing corrections were applied using the Benjamini and Hochberg method. Gene ontology/pathway enrichment analyses were carried out using Fatigo software [35], GeneSetDB [36], GATHER (http://gather.genome.duke.edu/) and IPA (Ingenuity systems, http://www.ingenuity.com).

### Apoptosis bayesian GRN generation and analysis

Apoptosis Bayesian networks were generated using the methods of [37], with some modifications. Given the relative sizes of the time-course and siRNA data sets, a dynamic GRN generated from the time-course data was used as a prior for GRN generated from the siRNA data as described [37].

When estimating the time-course GRN from the apoptosis time course microaray data, a method of bootstrapping was applied to the data. With 8 time points (obtained 0, 0.5, 1.5, 3, 6, 9, 12 and 24hrs after serum withdrawal) and 3 replicate microarray time course experiments, there are 3^8^ = 6561 possible combinations to create combinatorial apoptosis time course datasets. With such a large number of combinations, it is not computationally viable to fit regression curves through all combinations. Therefore the time course data used for network estimation was generated from the random resampling of 25 of the possible 6561 combinations as follows: Let *D* be the combinatorial time course data of all genes. If *D*(*c*) is the 8 time points, with each time point consisting of one of 3 replicates, then *D*(*c*) can be randomly resampled with replacement 25 times from the 6561 combinations so that *D*(*c*) (1 ≤ *c* ≤ 6561). The bootstrap sample can therefore be defined as D^*^ = {D^*^ (1),…., D^*^ (25)}. Using this sample of 8 x 25 = 200 randomly resampled microarrays, the apoptosis GRN was estimated. This bootstrapping procedure was repeated 100 times to generate 100 different GRNs; Ĝ^*^_  T_^    1^,… . Ĝ^*^_  T_^    100^, where Ĝ^*^_  T_^*    B*^ is the estimated graph based on the *B*-th bootstrap sample. To estimate the reliability of the edges to be used as prior information, the bootstrap probability of each edge was calculated as follows: the reliability of the edge between the *i*-th gene to the *j*-th gene (termed the bootstrap probability) is $z_{\mathrm{ij}}^{\left(1\right)}=|\left\{B|e\left(i,j\right)\in {\hat{G}}_{T}^{*B},B=1,\ldots ,100\right\}|/100$. A bootstrap probability threshold value was set at P = 0.8 and only those edges that passed this threshold value were included in the prior, *Z*_1_.

As described [37], a second prior (named the "array prior", Z_2_), was also generated. This prior was based on the up- or down-regulation of the abundance of all mRNAs, represented as z-scores, analysed by the microarrays following siRNA-medaited targeting of the 351 genes.

Priors *Z*_1_ and Z_2_, were used when inferring a static Bayesian network based on the disruptant dataset [37]. Again bootstrap resampling of the microarrays (100 times with replacement) was applied to improve the reliability of edges included in the final network. The GRNs were viewed and analysed using Cell Illustrator 5.0, freely available software that can be downloaded from http://www.cellillustrator.com.

### Quantification of apoptosis

Passage 3 HUVEC pools comprising equal numbers of cells from 10 independant isolates were plated at 5 × 10^3^ cells per well in a 96-well plate and cultured for 24 hrs before siRNA transfection. Three different pools of 10 isolates were used for each assay. Cells were then left for a further 24hrs before treatment with either survival factor deprived conditions of basal media without supplements (EBM-2) or fully supplemented media without antibiotics (EGM-2) for 24 hrs. Active caspase-3 and −7 were quantified using the Caspase-Glo 3/7 assay system, following the manufacturer’s instructions (Promega, Southampton, UK). The ADP:ATP ratio was calculated using the Apo Glow assay (Lonza, Cambridge, UK), according to the manufacture’s protocol. Assays were carried out using a Fluostar Optima luminometer (BMG Labtech, Aylesbury, UK). Statistical analysis was carried out using a paired two-tailed t-test.

## Results

### Gene selection methods for generating a bayesian GRN to model EC apoptosis

For GRN modelling, we first identified mRNA transcripts that were significantly regulated over the timecourse of EC apoptosis. A z-score-based method for analysis of timecourse data that we have reported previously ([18] and see Methods) identified 486 significantly regulated transcripts. We analysed these 486 RNAs using the GeneSetDB web tool [36] with the Gene Ontology (GO) and WikiPathways databases. The RNAs were significantly enriched for four main categories of annotation: (i) 'cell cycle' (GO:0007049, 87 RNAs, p<0.0001 and WikiPathways:WP179, 30 RNAs, p<0.0001), (ii) 'response to stress' (GO:0006950, 39 RNAs, p<0.0001), (iii) 'apoptosis' (Wikipathways:WP254, 7 RNAs, p=0.016) and (iv) 'immune response' (GO:0006955, 21 RNAs, p<0.0001).

### Bayesian GRN inference

**Figure 1:**
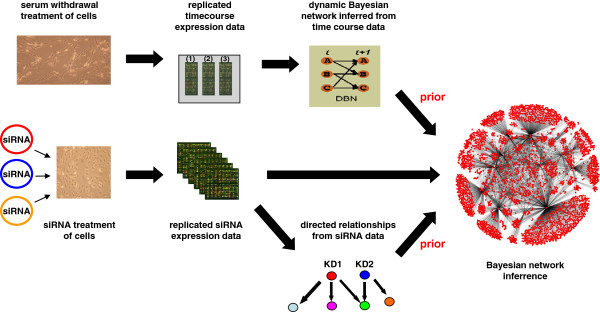
**Inference of a static Bayesian GRN.** Bayesian GRNs were generated from two microarray datasets (1) time course of primary ECs in conditions of SFD for 24 hours (8 time points in triplicate) to induce apoptosis and (2) disruptant dataset generated from the siRNA-mediated knockdown of 351 transcripts. These two datasets were used in network inference. Bayesian GRNs were generated to maximise the posterior probability, which consists of two priors; (a) the dynamic Bayesian GRN prior (generated from the time course data) and (b) the array prior (measuring the relationships between the gene knockdowns and their regulatees, as measured by z-score in the 351 disruptant dataset), as well as the marginal likelihood. This is the non-parametric regression through estimated edges based on the 351-disruptant dataset. The gene list of 694 transcripts chosen for network inference was selected based on (1) the transcripts regulated during the apoptosis time course and (2) the 351 siRNA targeted transcripts. Using the dynamic Bayesian GRN as a prior for the disruptant dataset, the relationships for the 694 transcripts within the 351 disruptant dataset were inferred. Bootstrapping of the network prior and the estimated static network helped improve edge reliability in the final network. The static apoptosis Bayesian GRN can be viewed using Cell Illustrator, which can be freely downloaded from http://www.cellillustrator.com.

In an attempt to better understand the relationships between the mRNAs concordantly regulated in abundance during SFD, a Bayesian GRN was inferred. In theory, the more data that GRN are based on, the the more accurate their predictions can be. Therefore, for GRN generation we used a combination of the SFD timecourse data and a library of 351 siRNA disruptant microarrays. A total of 694 RNAs were used for GRN generation; the union of the 486 RNAs concordantly regulated in abundance during SFD and the 351 RNAs that were targeted by siRNA. The methodology used to generate this Bayesian network has been previously described [37] and is illustrated in Figure 1. An xml file describing this GRN can be found in Additional file 1, which can be viewed using the freely available software Cell Illustrator, and a text file listing parent and child genes for the network edges is given in Additional file 2.

### Identification of GRN hubs

Hubs are highly connected nodes in GRNs and are candidate master regulators within the network structure. In a directional GRN such as the Bayesian networks generated here, they can be identified on the basis of having large numbers of downstream children. The distribution of the number of children of all nodes in the GRN is shown in the histogram in Additional file 3: Figure S1. A list of the 50 hub genes with the largest number of children (all with ≥20 children) in the network is shown in Additional file 4. Given that the set of RNAs used for GRN inference was already enriched for cell cycle and stress response functions as described above, it is not surprising that many of the GRN hubs appeared to be involved in these processes. Only 53 of the 351 genes that were targetted by siRNA knock-down to generate the prior microarray data were significantly concordantly regulated in abundance over the survival factor deprivation timecourse (according to the same z-score criteria that were used to select the 486 RNAs). Of these 53 RNAs, 10 are in the list of the to 50 hubs (ranked by the number of downstream children).

We looked specifically at apoptosis-associated RNAs in the GRN. Analaysis using the Fatigo software (http://www.fatigo.org/) identified 505 probes in the Codelink array data that encoded proteins involved in apoptosis, 58 of these probes were included as nodes in the apoptosis gene network (highlighted in Additional file 5); two of these (*HSPE1* and *BUB1B,* with 28 and 23 children respectively) were found in the top 50 hubs ranked by number of downstream network children.

We also looked specifically at cell cycle-associated RNAs in the GRN, since SFD induces cell cycle arrest in addition to programmed cell death [29]. Of the 596 transcripts on the Codelink array associated with cell cycle regulation, 109 of these were included as nodes in the network, with 9 located within the top 50 network hubs ranked by number of downstream children.

### Downstream children of some GRN Hubs share common functions

We assessed whether any of GRN hubs had downstream children significantly enriched for specific biological functions by comparing the downstream children of each hub to the datatbases GO, KEGG and Transfac using the GATHER web tool. The results of this analysis are shown in Additional file 4 and examples are given below. The hub transcript *BLM* (7^th^ largest hub, 33 children) encodes a DNA helicase that is important for mitotic DNA replication and DNA repair, and is mutated in a broad range of cancers [38]. 16 of the 31 children of *BLM* encoded proteins involved in aspects of cell cycle regulation (GO:0008283, GATHER Bayes Factor = 22.3, p = 3x10^-9^). Another hub gene encodes the cyclin dependent kinase-binding protein *CKS1B*, which has 16 of its 49 GRN children associated with cell cycle control (GO:0007049, GATHER Bayes Factor = 7.5 p = 0.007). The hub *KNTC2* encodes a kinetochore complex component that functions as a spindle checkpoint signalling molecule, and has 10 of its 18 children associated with the cell cycle (GO:0007049, GATHER Bayes Factor = 16 p = 7.5 × 10^-7^). Another hub is *GRN*, encoding the granulin glycoprotein, a secreted regulator of cell growth and survival. 10 of *GRN*’s 28 children are associated with the response to cell stress (GO:0006950, GATHER Bayes Factor = 8.6 p = 0.0005), and 5 with the regulation of apoptosis (GO:0042981, GATHER Bayes Factor = 5.3 p = 0.004). The children emenating from the apoptosis-associated hubs *HSPE1* and *BUB1B* mentioned above also shared comon functions, however the degree of enrichment for these functions was not statistically significant. Four of the 28 children of *BUB1B* were involved in cell cycle regulation (*CDC16, TPX2, NUSAP* and *BUB1*) and two in the regualtion of apoptosis (*PIK3R1* and *CTNAL1*). Similarly, three of the 23 children of *HSPE1* were involved in cell cycle processes (*RAD1, E2F4* and *MCM2*) and two with cell death (*TBP* and *FOSL2*).

### Identification of a novel GRN hub gene for further study

**Figure 2:**
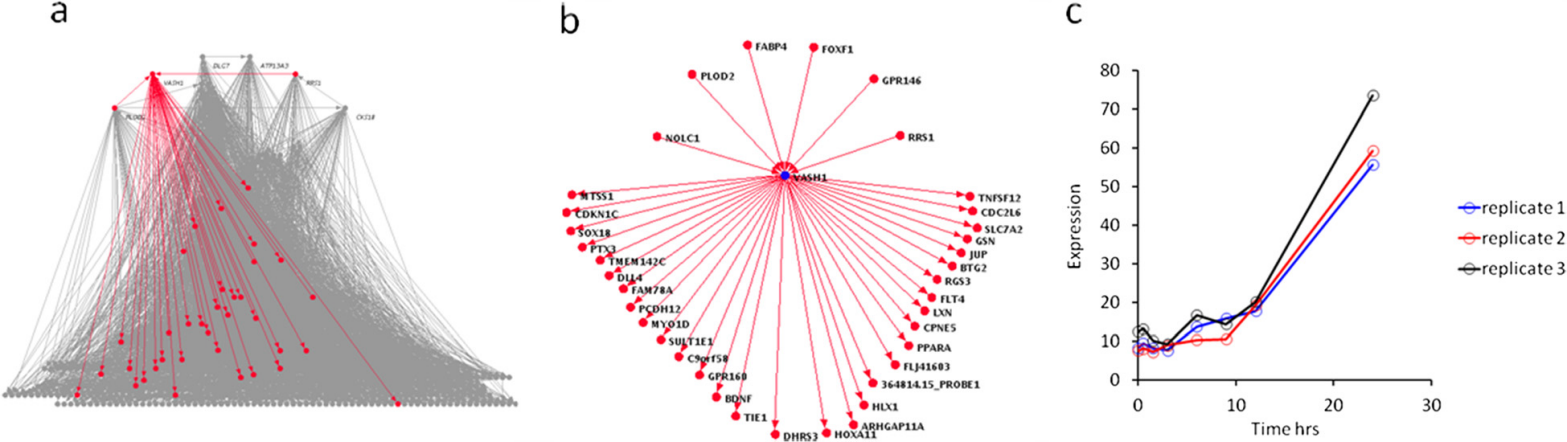
**The VASH1 hub in the Bayesian GRN.** The *VASH1* hub represents the 9^th^ largest hub in the GRN. (**a**) Positioning of the *VASH1* hub (highlighted in red) in the GRN topology. (**b**) Focussed analysis of the *VASH1* hub, illustrating the parents and children emanating from this hub gene. (**c**) The normlaised non-log transformed expression profile of *VASH1* mRNA in the three replicates across the SFD time course.

Evaluation of the literature revealed that several of the most highly connected hubs (such as *CKS1,* 49 children and *MDK,* 36 children), already had well-characterised roles in cellular proliferation and apoptosis [39-41]. In contrast, for *VASH1* (9^th^ ranked hub with 31 children) there was no literature characterising its involvement in EC apoptosis or cellular proliferation at the time of this study. Several of the predicted GRN children of *VASH1* are involved in the regulation of angiogenesis (e.g. *FLT4, BDNF, TIE1*), apoptosis (e.g. *TNFSF12, PPAR-α, CDC2L6*) and cell division (e.g. *BTG2, CDKN1C*). Therefore, given that the purpose of this current study was to identifiy novel regulators of EC apoptosis and since VASH1 had previously been identified as a negative regulator of angiogenesis [42,43] (suggesting a possible role in EC apoptosis), *VASH1* was selected as a candidate for further investigation. Due to resource limitations no other uncharacterised hubs were investigated in this study. Figure 2 illustrates the positioning of the *VASH1* hub in the GRN, the 31 children eminating from this hub, and its expression profile over the SFD time course.

*VASH1* would not have been prioritised as a candidate gene for further study by using traditional statistical methods that quantified the degree and variance of concordant regulation of abundance over the SFD time course. For example, when the concordant regulation of RNAs were ranked using the z-score method or using traditional ANOVA analysis to compare expression in the first and last timepoints to all other timepoints, *VASH1* was ranked 71st and 63rd, respectively. The empirical Bayes method of Tai and Speed using the Hotelling *T*^2^-statistic [34] ranked *VASH1* as 286th in terms of evidence of non-constant temporal expression. A more sophisticated method was also used, in which generalised estimating equations (with Markov correlation models) were fitted to the SFD time course data. From this regression model contrasts were used to identify linear relationships and quadratic trends within the data, and *VASH1* ranked 175th.

### Independent validation of directed edges emanating from the *VASH1* hub

**Figure 3:**
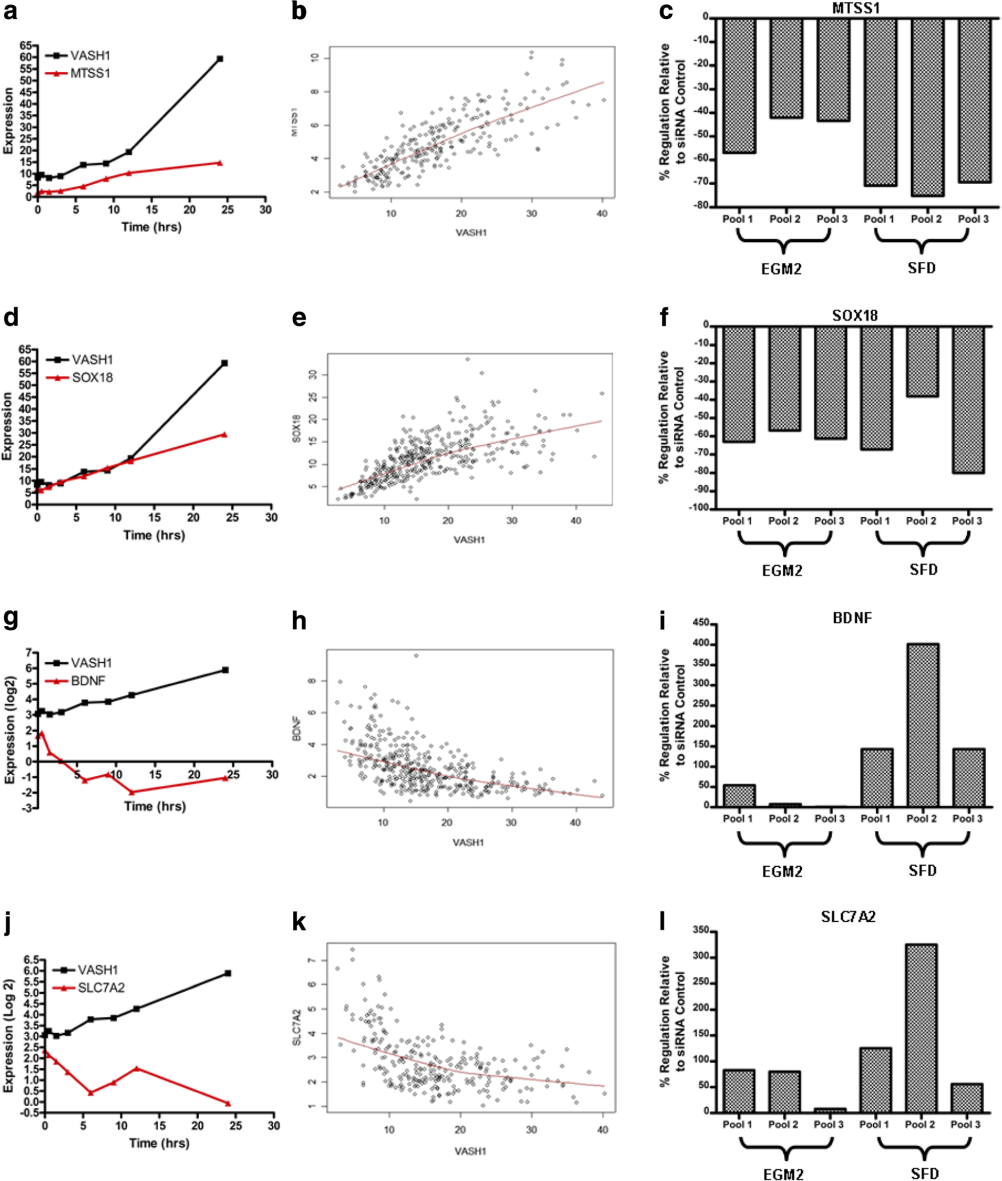
**Regulation of selected *VASH1 *predicted children (a) Co-regulation of predicted child *MTSS1 *with *VASH1 *over the median expression value of the triplicate apoptosis time course (b) Correlation of predicted child *MTSS1 *expression with *VASH1 *across the 351 disruptant dataset (c) Relative level of predicted child MTSS1 mRNA when *VASH1 *mRNA abundance is knocked down to ≤ 20% of its initial value.** The knockdown of VASG1 was carried out in both fully supplemented conditions (EGM2) and survival factor deprived conditions (SFD), to assess the impact of the knockdown in both conditions. (**d – f**) as above for predicted child *SOX18*. (**g – i**) as above for predicted child BDNF. (**j – l**) as above for predicted child *SLC7A2.*

To evaluate the RNAs hypothesised by the GRN to be downstream of the *VASH1* hub, 10 of the 31 children were selected for independent validation using siRNA knock-down and quantitative PCR. The selection was based on known biological importance and reagent availability. The left column of panels in Figure 3 illustrates the transcript profiles of *VASH1* and selected children over the SFD time course. In the case of *MTSS1* (3a) and *SOX18* (3d), the children are positively co-regulated with *VASH1* over the apoptosis time course. In contrast, *BDNF* (3g) and *SLC7A2* (3j) are negatively co-regulated with *VASH1* over the time course. Correlation analysis across the 351 siRNA disruptant dataset revealed that all 10 children correlated with *VASH1* (correlation coefficients range from 0.5 – 0.8); the relationships between *VASH1* and these downstream children across the 351 siRNA disruptant microarrays are illustrated in scatter plots in the middle panels of Figure 3. *VASH1*-*SLC7A2* and *VASH1-BDNF* associated with negative correlation, while the remaining children correlate positively with *VASH1* (Figure 3b, e, h and k). This correlation across the 351 siRNA disruptant dataset concurred with the co-regulation observed over the apoptosis time course in Figure 3a, d, g and j.

Using siRNA to knockdown *VASH1* mRNA to ≤ 20% of its initial level appeared to significantly regulate 7 of the 10 transcripts tested, in the direction predicted by the GRN (Table 1). For example, *MTSS1* and *SOX18*, which were positively correlated with *VASH1*, were down-regulated after knockdown of *VASH1* (Figure 3c and 3f respectively). In contrast, but as predicted by the GRN, knockdown of *VASH1* resulted in the up-regulation of *BDNF* and *SLC7A2*, which were negatively correlated with *VASH1* (Figure 3i and Figure 3l). *TNFSF12*, *PTX3* and *FAM78A* did not show a clear result due to variable regulation between EC replicate pools.

**Table 1:** **Fold change and P values of *VASH1 *and its predicted children in fully supplemented conditions and SFD conditions, after knockdown of *VASH1***

	**Fully Supplemented Media (EGM2)**		**Survival Factor deprived Media (SFD)**	
	**Fold Change**	**P Value**	**Fold Change**	**P Value**
**VASH1**	−7.59	0.0022	−17.50	0.0025
**BDNF**	3.90	0.4138	12.52	0.0339
**BTG2**	−1.65	0.1592	−3.10	0.0005
**FAM78A**	−1.97	0.0577	−0.69	0.5752
**FLT4**	−1.99	0.0404	−3.67	0.0364
**MTSS1**	−1.82	0.0128	−3.97	0.0708
**PPARA**	−1.92	0.0962	−2.79	0.0034
**PTX3**	−1.04	0.4932	0.54	0.6942
**SLC7A2**	1.96	0.1087	2.30	0.0237
**SOX18**	−1.41	0.5587	−3.81	0.0002
**TNFSF12**	0.46	0.5499	−1.82	0.0223

P values are from a paired t test.

**Figure 4:**
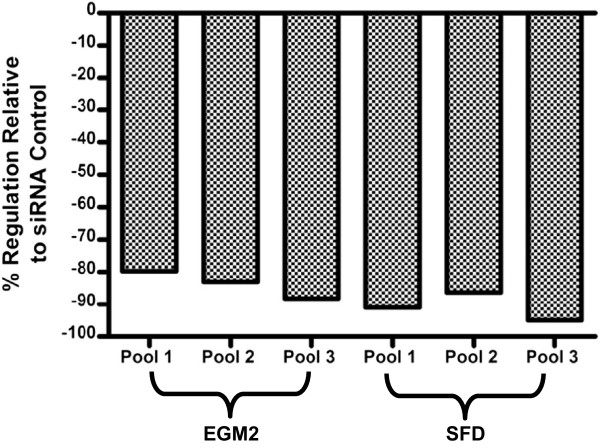
Quantitative PCR of siRNA-mediated knockdown of VASH1 mRNA abundance to <20% of its initial level in three HUVEC pools in both fully supplemented media conditions (EGM2) and survival factor deprived conditions (SFD).

### Regulation of apoptosis by *VASH1*

**Figure 5:**
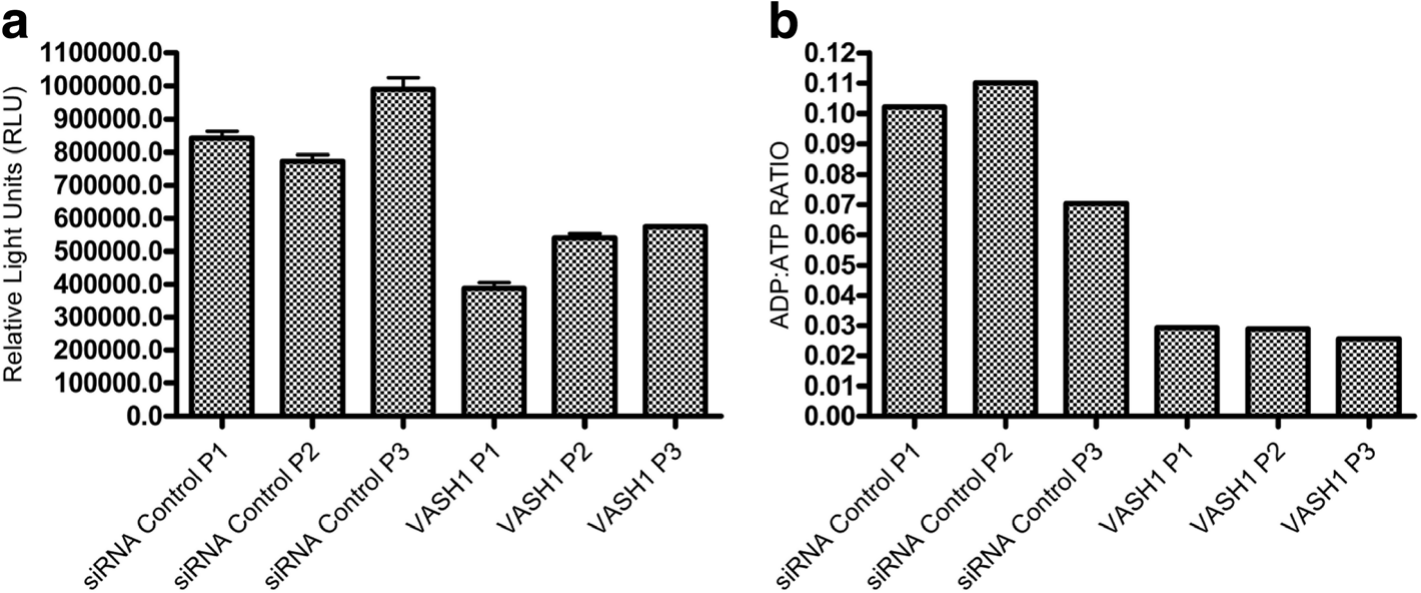
**(a) Quantification of active caspase-3 and −7 in three independent pools of 10 HUVEC isolates treated with either a non-targeting siRNA for 48hrs or siRNA against *VASH1 *for 48 hrs.** HUVECs were treated 24hrs post transfection with survival factor deprived (SFD) conditions for 24hrs before measurement. A significant difference was observed between the VASH1 knockdown and the siRNA control in the SFD condition (P = 0.0009, paired two tailed t-test). (**b**) Quantification of the ADP:ATP ratio in three pools of 10 HUVEC isolates treated with either a non-targeting siRNA for 48hrs or siRNA against *VASH1* for 48hrs. HUVECs were treated 24hrs post transfection with SFD conditions for 24hrs before measurement (P = 0.02). P = Pool.

To evaluate whether the *VASH1* hub is involved in the process or regulation of EC apoptosis, siRNA was used to knockdown *VASH1* in three different pools of 10 HUVEC isolates for 24 hours before treatment with SFD to induce apoptosis. After the 24 hour anti-*VASH1* siRNA incubation, *VASH1* mRNA abundance was reduced to ≤ 20% of its initial level (Figure 4). Following SFD there was a mean of 2.2 fold (t-test, p = 0.0009) less active caspase-3 and −7 in the *VASH1* knockdown EC compared to the EC teated with non-targeting siRNA controls (Figure 5a). Repetition of this assay in two additional pools of HUVEC isolates in which *VASH1* was once again knocked down to ≤ 20% of its initial level produced a similar result - following SFD there was on average 1.8 fold (p = 0.03) less active caspase-3 and −7 following *VASH1* knockdown than in control cells (data not shown). The observed level of active caspase-3 and −7 in HUVEC in fully supplemented conditions was similar in VASH1 knockdown and control cells (data not shown).

The activation of caspase-3 and −7 only represents one part the complex process of apoptosis. Since apoptosis is an energy driven process, the ADT:ATP ratio was also calculated in the same HUVEC pools. A marked reduction in the mean ADP:ATP ratio was observed in the *VASH1* knockdown EC relative to the siRNA control EC following SFD in two independent experiments (12.4 fold, P=0.004 and 3.4 fold, P=0.005, respectively, Figure 5b). Again, no significant difference was observed between the *VASH1* knock down EC and controls in fully supplemented conditions (data not shown). Taken together, these results suggest that VASH1 may play a significant role in SFD-induced apoptosis of HUVECs.

The inverse expression relationship between *VASH1* and its validated child *BDNF*, and the known role of BDNF as a survival factor, suggests the hypothesis that up-regulation of *BDNF* when *VASH1* is knocked down may promote survival in these cells. However, the treatment of the HUVEC pools with 100nM recombinant *BDNF* at 24 hours post-transfection (at the same point as treatment with either fully supplemented media or SFD conditions), did not induce significant rescue from SFD-induced cell death, as measured by both the quantification of active caspase-3 and 7 and the ADP:ATP ratio (data not shown).

## Discussion

This study used Bayesian GRN technology and microarray data to model the regulatory interactions after serum factor deprivation of EC, which induces cell cycle arrest and apoptosis. We applied GRN analysis to combined time course microarray data following serum deprivation-induced apoptosis of EC, and a large microarray data set generated from 351 targeted EC siRNA disruptions, in order to identifiy new information about gene regulatory relationships during EC apoptosis.

### GRN topology and limitations

We have used GRN analysis to identify hubs, which potentially act as master regulators of the expression of downstream RNAs in EC. For one hub, *VASH1*, we then confirmed the concordant regulation of a subset of downstream children as hypothesised by the GRN, and an impact on apoptosis. VASH1 protein has previously been identified as a negative feedback regulator of angiogenesis, and is induced through signalling via VEGFR2 and protein kinase C [43,44]. These findings are consistent with a role for VASH1 as a key regulator of EC biology, and with the position of *VASH1* as a hub in the GRN with an effect on EC apoptosis.

We suggest that GRN analysis may provide a useful compliment to traditional analysis of microarray or RNAseq data, especially for identifying putative master-regulators for further study. We showed that alternative methods to identify apoptosis-associated RNAs from time course expression data were unlikely to have selected *VASH1* as a candidate gene for subsequent functual analaysis. Methods based on: z-scores, ANOVA and empirical Bayes all failed to prioritise *VASH1*. However, we also recognise the potential limitations of gene network analysis. Firstly, only a few of the transcripts that encode protein mediators of apoptosis are expected to be up- or down-regulated during relatively short time points of SFD-induced apoptosis [29] to a sufficint degree to be included in this analysis, and SFD is only one of many inducers of apoptosis. Therefore, only a subset of known apoptosis-associated genes are accessible to this type of GRN study. Secondly, many genes known to be important in the apoptotic process may not be “master regulators” detectable as hubs in gene networks, which requires that they rapidly regulate the abundance of large numbers of downstream RNAs. Since we used timecourse microarray data from EC cells treated with survival factor deprivation, we expected to identify only master regulators that were specifically related to the molecular processes that occur during this timecourse. To be identified as a "hub" in the gene network, both an RNA, and its downstream "child" RNAs, must be significantly concordantly regulated over the timecourse. Therefore, not all RNAs important for EC biology will be concordantly regulated during this specialised type of apoptosis, and even if they are, they will not be identified as hubs unless they rapidly regulate the abundance of large numbers downstream RNA transcripts. Thirdly, like all *in silico* modelling based on microarray or RNAseq data, results of special interest from GRN analysis need to be confimred using laboratory experiments as we have done here.

### Inference of local relationships within the network

Several GRN methods have proven informative for identifying regulatory hubs or cohorts of co-expressed genes in complex eukaryotic cells, which are involved in important disease processes [10,45-47]. However, most of these methods fall short of inferring directional relationships at a local level. Therefore, having identified the *VASH1* hub based on network topology, we examined the GRN predictions surrounding this hub in more detail. Using siRNA we knocked down *VASH1* mRNA and determined the effect on expression levels of downstream mRNAs for ten out of *VASH1*'s 31 GRN children. Seven out of the ten children tested were significantly up- or down-regulated in the direction predicted by the GRN (see Table 1). The lack of clear influence of *VASH1* knock-down on three child-transcripts may be due to several factors: (i) Reducing *VASH1* RNA may have little effect on the abundance of those gene network children of *VASH1* that are strongly influenced by other parents in addition to *VASH1* - the undiminished effects of these other parents would be expected to hide the effect of reducing *VASH1* expression. (ii) Regulatory relationships that are not represented in the GRN may influence the expression of some of *VASH1*'s gene network children, (iii) Despite best efforts, the effects of experimental noise and unintended model over-fitting are likely to have introduced error in the inference process. These issues are further described in our recent publications [48,49]. It is possible that additional siRNA data may improve the accuracy of GRNs around *VASH1*, which is a subject for future research.

Whether the observed level of concordance between the network predictions and the results of experimental *VASH1* knockdown only surrounds the major hubs within the Bayesian network structure, or is randomly distributed throughout the network, requires further investigation. Due to resource constraints we have only evaluated a minority of edges downstream of a single hub. This is clearly not enough to draw any general conclusions about GRNs and their reliability. Given further resources, we would like to evaluate the relationships between *VASH1* and the remaining 21 children that we have not yet tested, as well as the relationships between several other nodes and their children. To more completely test local network relationships we would ultimately need to simultaneously perform siRNA-mediated "knockdown" of all the gene network parents of each *VASH1* child then measure the effect on *VASH1* child abundance. In addition, as computational capabilities improve, it would be interesting to re-engineer GRNs with the inclusion of more of the replicate arrays and compare the reliability with that of the current network model. Nevertheless, given the early stage of this technology, the fact that only one (*VASH1*) of several parents of the evaluated *VASH1* children was knocked down, and the fact that there are data missing from the network (due to computational constraints it contains only 694 transcripts), these current findings appear promising.

### Functional significance of the VASH1 gene network hub

To investigate whether the *VASH1* hub was biologically relevant during endothelial apoptosis, we used siRNAs targeted against *VASH1* to reduce mRNA abundance to <20% of its initial level and quantified the level of apoptosis in these cells under conditions of SFD relative to control cells transfected with an irrelevant siRNA. The measurement of active caspase-3 and −7 and the ADP:ATP ratio were used as end-points and the knock-down of *VASH1* conferred resistance to the pro-apoptotic stimulus of serum deprivation. This confirms a role for *VASH1* in the process of EC apoptosis, and is consistent with a study published since this research was conducted that shows over expression of VASH1 induced apoptosis in proliferating human fibroblasts [50].

Although the mechanism by which VASH1 regulates EC survival is beyond the scope of this study, it is intriguing to examine the function of *VASH1*’s GRN children in the anticipation that this may suggest how VASH1 acts. Ingenuity Pathways Analysis (IPA) suggested that the *VASH1* GRN children are significantly enriched for genes associated with angiogenesis (FDR = 0.025), including several well-known angiogenic regulators; *BDNF*, *DLL4* (*delta-like 4*), *FLT4* (*vegfr-3*), *PPARA*, *PTX3*, *SOX18*, *TIE1*, and *TNFSF12* (*tweak*). Several of the *VASH1* children have previously been associated with the linked control of proliferation and apoptosis e.g. *CDKN1C* (p57/kip2), *CDC2L6*, *GSN* (*gelsolin*) and *DLL4*. It is interesting that 20 of the 31 *VASH1* children have binding sites for members of the E2F transcription factor family in their promoters (V$E2F1_Q3, FDR = 0.002); experiments to assess whether E2F transactivation is regulated by VASH1 may be worthwhile.

One mechanistic hypothesis was investigated in this study. The autocrine action of BDNF in cellular processes including angiogenesis, proliferation, differentiation and survival is well documented [51-53]. Therefore, the inverse relationship between *VASH1* and its validated child *BDNF*, suggests a hypothesis that upregulation of *BDNF* when *VASH1* is knocked down may promote survival. However, recombinant *BDNF* was unable to rescue the cells from SFD induced death. It is possible that the chosen concentration of recombinant BDNF (100nM) and time point of administration were not optimal. However, the results suggest that it is unlikely that the mechanism of *VASH1* action in EC apoptosis is as simple as up-regulation of *BDNF.* The relatively low expression of the BDNF high affinity (TrkB) and the low affinity (p75^NTR^) receptors observed in the microarray data from these cells (*TrkB*, percentile rank expression = 0.142113 and *p75*^*NTR*^, percentile rank expression = 0.414449 in healthy HUVEC) may also explain why exogenous BDNF was not effective.

Since this study was conducted, several publications have described the role of *VASH1* in regulating EC proliferation, vascular tube generation and vascular maturation [50,54][55], as well as a potentially wider functional role in other cell types [56]. In addition, VASH1 may interact in complex functional networks with related proteins such as VASH2 to regulate angiogensis and EC survival differently in distinct angiogenic mechanisms [57]. Whatever its mechanism of action, VASH1 appears to be associated with angiogenesis in pathology [43,58], and further investigation of the molecular networks that surround *VASH1* seem highly worthwhile.

## Conclusion

GRN analysis is able to supplement the reductionist methods of traditional molecular biology, providing testable hypotheses about the synergistic actions and interactions of multiple molecules [59]. We therefore applied Bayesian GRN methods to further our understanding of the regulation of EC apoptosis and proliferation. The SFD Bayesian GRN generated in this study identified *VASH1* as a candidate master regulator, which we found was functionally important during EC apoptosis. We also found that several individual directed edges emanating from *VASH1* in the GRN appeared to operate in ECs. We hope that in future studies the datasets we describe here can be used by other researchers to identify additional candidate master regulators for laboratory evaluation.

## Competing interests

Ther are no strong competing interests. However, for completenes we wish to disclose that: HA, YT and CS are past employees of GNI Ltd., a Japanese biotechnology company which supported the generation of microarray data used in this study. All authors have held stock or stock options in GNI Ltd. None of the authors are current employees of GNI Ltd.

## Authors’ contributions

All authors fullfil the criteria for authorship. MA wrote the manuscript and primarily generated the new experimental data shown here. DS, SH and BD collaborated extensively with and supervised MA in the laboratory for molecular biology and microarray studies and assisted with data analysis. HA undertook bioinformatics of siRNA array data and assisted with Bayesian network analysis. YT and SI performed Bayesian network analysis and gave advice on network inference issues. CS, SM and SK assisted with the planning and funding of the project, supervised the generation of gene network software and its use. DJ performed statistical analysis and provided statistical advise. CP and DSCJ jointly supervised MA as a postgraduate student, undertook the experimental planning of the project, oversaw and assisted with data interpretation and manuscript preparation.

## Authors’ information

Cristin Print and Stephen Charnock-Jones Joint senior authors.

## Supplementary Material

Additional file 1 XML file describing the GRN. Click here for file

Additional file 2 List of the parent child relationships inferred from the GRN. Click here for file

Additional file 3: Figure S1 The histiogram shows the frequency (y-axis) of number of children (x-axis) in the GRN. Click here for file

Additional file 4 List of the 50 hub genes with the largest number of children in the GRN. Click here for file

Additional file 5 List of apoptosis associated RNA’s in the Codelink microarray platform and the GRN. Click here for file
