# Supplementary figures and images for: Vasohibin-1 is identified as a master-regulator of endothelial cell apoptosis using gene network analysis
Source: BMC Genomics. 2013 Jan 16;14:23. doi: 10.1186/1471-2164-14-23 (PMC3570387; doi:10.1186/1471-2164-14-23)

## Slide 1
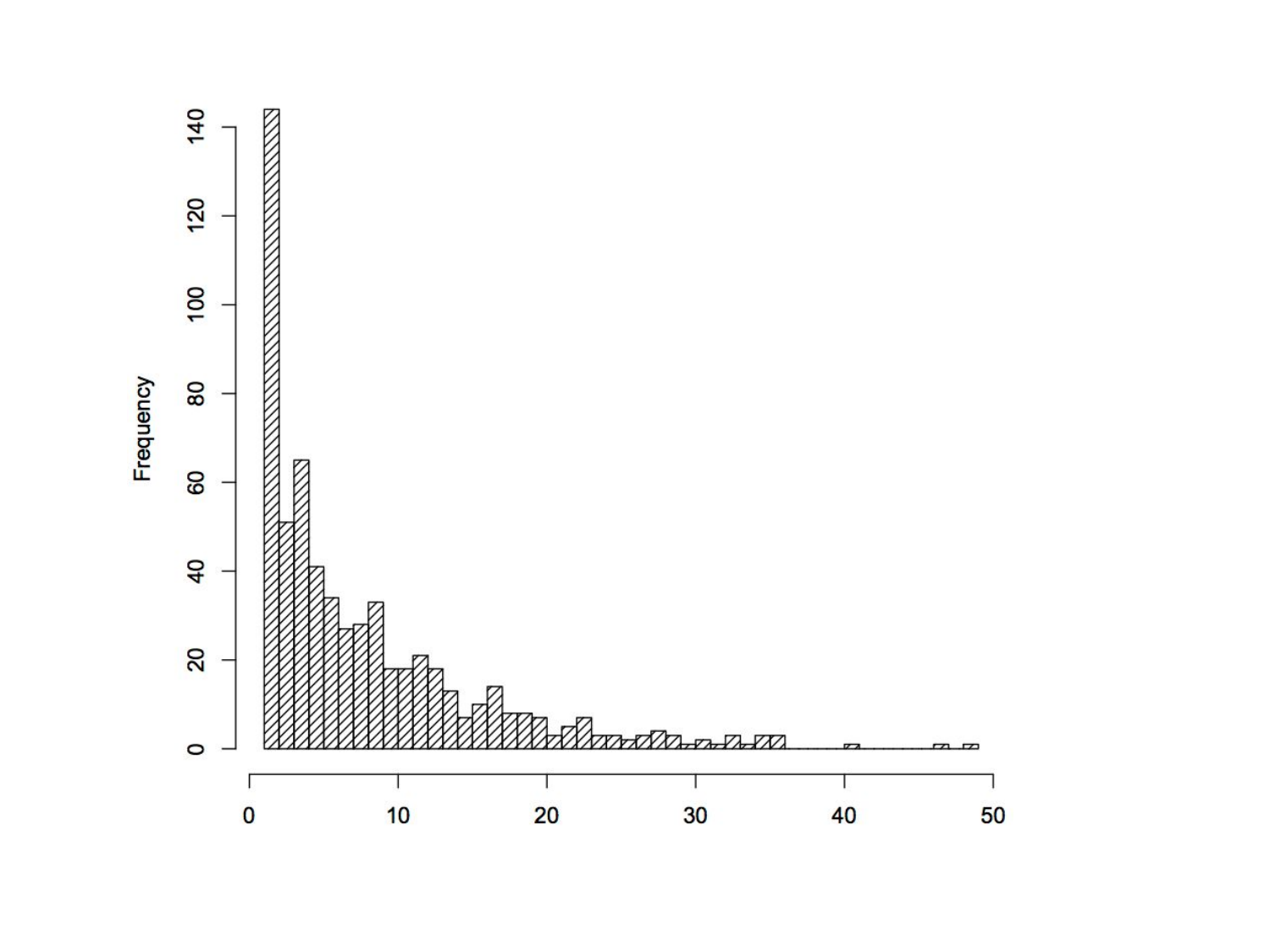

Supplement: Additional file 3: Figure S1 — The histiogram shows the frequency (y-axis) of number of children (x-axis) in the GRN. [file 1471-2164-14-23-S3.ppt]
