# Supplementary material for: Vasohibin-1 is identified as a master-regulator of endothelial cell apoptosis using gene network analysis
Source: BMC Genomics. 2013 Jan 16;14:23. doi: 10.1186/1471-2164-14-23 (PMC3570387; doi:10.1186/1471-2164-14-23)
Supplement: Additional file 4 — List of the 50 hub genes with the largest number of children in the GRN. [file 1471-2164-14-23-S4.doc]

| **Gene Name** | **CodeLink ProbeID** | **Children** | **Parents** |
| --- | --- | --- | --- |
| CKS1B | 1501987.1_PROBE1 | 49 | 2 |
| ATP13A3 | 1454113.6_PROBE1 | 47 | 7 |
| DLG7 | NM_014750.1_PROBE1 | 41 | 2 |
| MDK | 1681813CB1_PROBE1 | 36 | 7 |
| PLOD2 | NM_000935.1_PROBE1 | 36 | 8 |
| KIF4A | NM_012310.2_PROBE1 | 36 | 8 |
| TPX2 | NM_012112.1_PROBE1 | 35 | 7 |
| FLJ20105 | NM_017669.1_PROBE1 | 35 | 5 |
| PLK4 | NM_014264.1_PROBE1 | 34 | 4 |
| BLM | NM_000057.1_PROBE1 | 33 | 1 |
| EXO1 | NM_006027.1_PROBE1 | 33 | 2 |
| SPAG5 | NM_006461.1_PROBE1 | 33 | 4 |
| RRS1 | NM_015169.1_PROBE1 | 32 | 6 |
| VASH1 | NM_014909.1_PROBE1 | 31 | 6 |
| CCDC99 | NM_017785.1_PROBE1 | 31 | 9 |
| ETS2 | 1998155CB1_PROBE1 | 30 | 4 |
| UBE2C | NM_007019.1_PROBE1 | 30 | 7 |
| YPEL3 | 1375644CB1_PROBE1 | 29 | 3 |
| TRAIP | NM_005879.1_PROBE1 | 29 | 3 |
| POLQ | NM_006596.1_PROBE1 | 29 | 2 |
| HSPE1 | 1226628CB1_PROBE1 | 28 | 8 |
| IL2RG | 1503691.2_PROBE1 | 28 | 5 |
| POLR3G | 980742CB1_PROBE1 | 28 | 3 |
| GRN | NM_002087.1_PROBE1 | 28 | 7 |
| CXCR3 | 332177.1_PROBE1 | 27 | 5 |
| TOPBP1 | NM_007027.1_PROBE1 | 27 | 5 |
| SPBC25 | NM_020675.1_PROBE1 | 27 | 5 |
| CXCL11 | 2768553CB1_PROBE1 | 26 | 22 |
| C10orf11 | NM_032024.1_PROBE1 | 26 | 6 |
| LYN | NM_002350.1_PROBE1 | 25 | 5 |
| GABARAP | NM_007278.1_PROBE1 | 25 | 9 |
| EXOSC2 | NM_014285.1_PROBE1 | 25 | 5 |
| FLJ41603 | AK023814_PROBE1 | 24 | 6 |
| PRPS1L1 | NM_002764.1_PROBE1 | 24 | 7 |
| SPRY2 | NM_005842.1_PROBE1 | 24 | 5 |
| RFC4 | 1729693CB1_PROBE1 | 23 | 10 |
| IL4I1 | 404400.3_PROBE1 | 23 | 9 |
| FST | 8048114CB1_PROBE1 | 23 | 6 |
| GPR146 | 982901.6_PROBE1 | 23 | 2 |
| BUB1B | NM_001211.2_PROBE1 | 23 | 5 |
| DKFZp762E1312 | NM_018410.1_PROBE1 | 23 | 5 |
| LXN | 1649584CB1_PROBE1 | 22 | 5 |
| IL15 | 2469073CB1_PROBE1 | 22 | 4 |
| RRM2 | NM_001034.1_PROBE1 | 22 | 5 |
| TUBG2 | NM_001070.1_PROBE1 | 22 | 7 |
| C15orf20 | 203678.1_PROBE1 | 21 | 6 |
| PRIM1 | NM_000946.1_PROBE1 | 21 | 8 |
| ETV7 | NM_016135.1_PROBE1 | 21 | 14 |
| 1386170.1_PROBE1 | 1386170.1_PROBE1 | 20 | 2 |
| PTX3 | 1966280CB1_PROBE1 | 20 | 4 |

**Supplementary file 2:** Table of top 50 hubs in the gene regulatory network, based on the number of children.
